# Anti-Inflammatory Effect of Wogonin on RAW 264.7 Mouse Macrophages Induced with Polyinosinic-Polycytidylic Acid

**DOI:** 10.3390/molecules20046888

**Published:** 2015-04-16

**Authors:** Ji Young Lee, Wansu Park

**Affiliations:** Department of Pathology, College of Korean Medicine, Gachon University, Seongnam 461-701, Korea; E-Mail: oxygen1119@hanmail.net

**Keywords:** wogonin, dsRNA, inflammation, macrophages, nitric oxide, cytokine, calcium, STAT1, STAT3

## Abstract

Wogonin (5,7-dihydroxy-8-methoxyflavone) is an active flavonoid compound originally isolated from *Scutellaria radix*, which has been used to treat lung inflammation in Korea, China, and Japan. Wogonin has been known to inhibit inducible nitric oxide synthase and have the anti-tumor properties. However, the effects of wogonin on virus-induced macrophages are not fully reported. In this study, the anti-inflammatory effect of wogonin on double-stranded RNA (dsRNA)-induced macrophages was examined. Wogonin restored the cell viability in dsRNA [polyinosinic-polycytidylic acid]-induced RAW 264.7 mouse macrophages at concentrations of up to 50 μM. Wogonin significantly inhibited the production of nitric oxide, IL-1α, IL-1β, IL-6, IL-10, IP-10, G-CSF, GM-CSF, LIF (IL-6 class cytokine), LIX/CXCL5, MCP-1, M-CSF, MIP-1α, MIP-1β, MIP-2, RANTES/CCL5, TNF-α, and VEGF as well as calcium release and mRNA expression of signal transducer and activated transcription 1 (STAT1) and STAT3 in dsRNA-induced RAW 264.7 cells (*P* < 0.05). In conclusion, wogonin has anti-inflammatory properties related with its inhibition of nitric oxide, cytokines, chemokines, and growth factors in dsRNA-induced macrophages via the calcium-STAT pathway.

## 1. Introduction

Inflammation is part of the non-specific immune response that occurs in reaction to harmful stimuli such as pathologic microbes, damaged cells, irritants, or any type of bodily injury, with a primary aim of neutralizing infectious agents and initiating repair to damaged tissue [1]. But the uncontrolled inflammatory phenomena could be a turning point for the development of acute or chronic inflammatory diseases. Thus, till now, the regulation of inflammation is the important medical interest. Among the many immuno-inflammatory leukocytes, macrophages and monocytes are of great importance [2].

Phagocytes, including monocytes and macrophages, play a crucial role in host defense by mediation of crucial physiological and protective functions such as innate immunity and inflammatory reactions, elimination of invading pathogens, and scavenging dead cells [3].

Macrophages play an important role in inflammatory disease through the release of factors such as nitric oxide (NO), reactive oxygen species, inflammatory cytokines, chemokines, growth factors, and prostaglandin mediators involved in the immune response [4].

NO is essential for host innate immune responses to pathogens such as viruses, bacteria, fungi, and parasites [5]. Sustained production of NO endows macrophages with cytostatic or cytotoxic activity against viruses, bacteria, fungi, protozoa, helminths, and tumor cells [6]. However, excessive production of NO is related with the development of septic shock, neuropathological diseases, rheumatoid arthritis (RA), and other autoimmune disorders [7].

Cytokines including chemokines and growth factors are soluble proteins that are secreted by cells of the immune system. They can alter properties of different cell types and provide essential communication signals for motile cells of the immune system [8]. Chemokines and their receptors play a central role in the inflammatory recruitment of leukocytes and other cell types [9]. But, excessive and uncontrolled production of these inflammatory cytokines may lead to serious systemic complications such as microcirculatory dysfunction, tissue damage, and septic shock, which can exact a high mortality [10].

**Figure 1:**
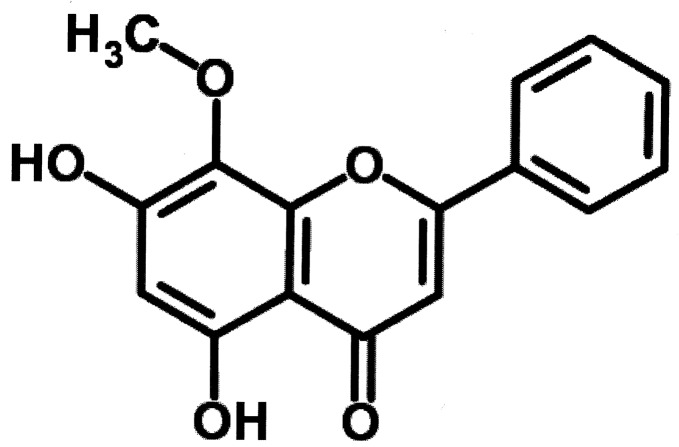
Structural formula of the flavonoid wogonin.

Wogonin (5,7-dihydroxy-8-methoxyflavone; Figure 1) is an active flavonoid compound originally isolated from *Scutellaria radix*, which has been used to treat lung inflammation in Korea, China, and Japan. Wogonin has been known to inhibit inducible nitric oxide synthase and have anti-tumor property [11]. However, the effects of wogonin on virus-induced macrophages are not fully reported.

The double-stranded RNA (dsRNA), which accumulates at various stages of viral replication, stimulates macrophages [12]. Polyinosinic-polycytidylic acid (PIC) is considered as the synthetic analog of dsRNA. Like other pathogenic endotoxin, dsRNA-activated macrophages provokingly produce many kinds of inflammatory mediators, including NO, cytokines, chemokines, and growth factors, resulting in acute or chronic inflammation [12].

We investigated the inhibitory effects of wogonin on PIC-induced inflammation using RAW 264.7 mouse macrophages. Finally, it was demonstrated that wogonin inhibits the excessive production of NO, interleukin (IL)-1α, IL-1β, IL-6, IL-10, interferon inducible protein-10 (IP-10), monocyte chemotactic protein (MCP)-1, granulocyte colony-stimulating factor (G-CSF), granulocyte macrophage colony-stimulating factor (GM-CSF), leukemia inhibitory factor (LIF; IL-6 class cytokine), lipopolysaccharide-induced CXC chemokine (LIX; CXCL5), macrophage colony- stimulating factor (M-CSF), macrophage inflammatory protein (MIP)-1α, MIP-1β, MIP-2, RANTES/CCL5, tumor necrosis factor (TNF)-α, and vascular endothelial growth factor (VEGF) as well as calcium release and mRNA expression of signal transducer and activated transcription 1 (STAT1) and STAT3 in PIC-induced RAW 264.7 mouse macrophages.

## 2. Results and Discussion

### 2.1. Effects of Wogonin on Cell Viability

**Figure 2:**
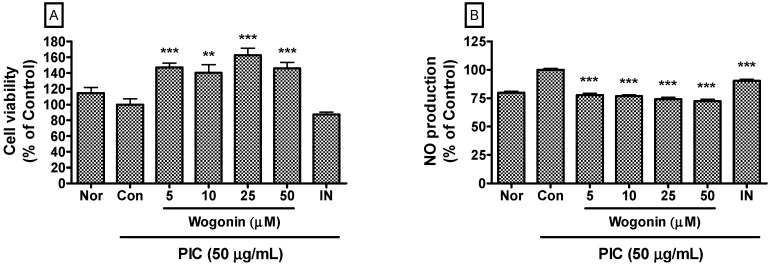
Effects of wogonin on cell viability and NO production in dsRNA-induced RAW 264.7 mouse macrophages. After 24 h treatment, cell viability (**A**) was evaluated by a modified MTT assay and NO production (**B**) was measured by the Griess reaction. Normal group (Nor) was treated with media only. Control group (Con) was treated with the synthetic analog of dsRNA (PIC, 50 µg/mL of polyinosinic-polycytidylic acid) alone. IN denotes indomethacin (0.5 µM). Values are the mean ± SD of three independent experiments. ** *P* < 0.01 *vs.* Con; *** *P* < 0.001.

Tissue resident macrophages are among the first cells to detect microorganisms that have crossed an epithelial barrier. They then recruit large numbers of neutrophils, followed by blood monocytes that differentiate into macrophages upon entry into the affected tissue [13,14,15]. It was already known that pathogens of infectious diseases reduce the cell viability of macrophages. In this study, wogonin up to a concentration of 50 µM restored the cell viability in PIC-induced RAW 264.7 mouse macrophages. With this result, wogonin concentrations of up to 50 µM were chosen for subsequent experiments (Figure 2A).

### 2.2. Effects of Wogonin on NO Production

Data represented that wogonin significantly inhibits excessive production of NO in PIC-induced RAW 264.7 mouse macrophages (*P* < 0.05) (Figure 2B). Ungoverned inflammation is an underlying component of various diseases, such as sepsis, cardiovascular disease, diabetes, and other chronic inflammatory diseases [16]. In the case of viral infection, double-stranded RNA of pathogenic viruses is the strong initiator of inflammation and stimulates macrophages to produce inflammatory mediators including NO, cytokines, chemokines, and growth factors in immune responses and organ injuries [12]. In the development of multiple organ failure, excess NO production caused by infections is implicated with a putative mechanism involving direct mitochondrial inhibition, predominantly affecting complex I [17]. In the addition, overproduced NO causes vasodilatation, hypotension, vascular leakage, and disruption of cell metabolism [18]. Thus, wogonin could be a candidate material for relieving viral inflammatory diseases concerned with excessive NO production.

### 2.3. Effects of Wogonin on Cytokine Production

**Figure 3:**
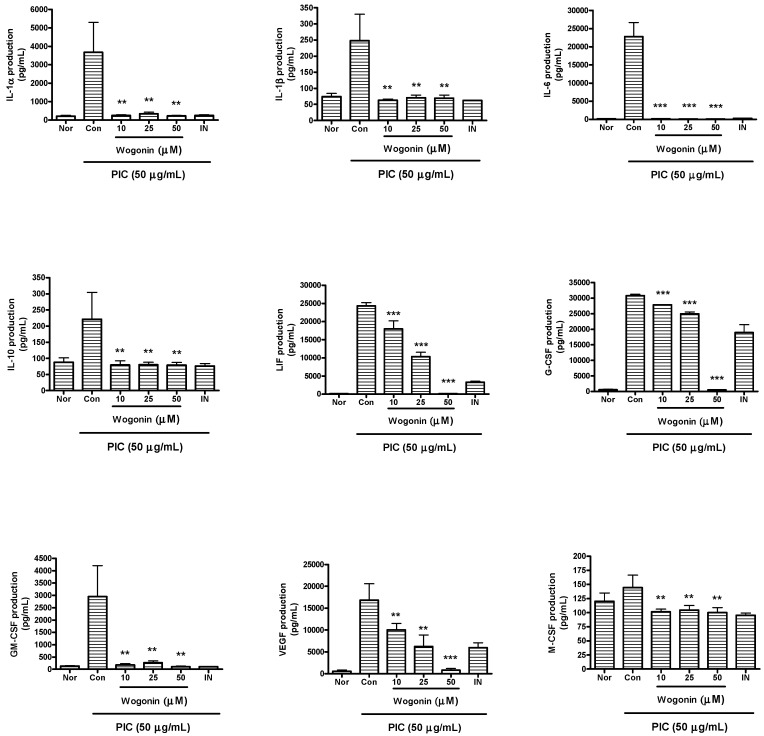
Effects of wogonin on production of cytokines such as IL-1α, IL-1β, IL-6, IL-10, LIF, G-CSF, GM-CSF, VEGF, and M-CSF in dsRNA-induced RAW 264.7 mouse macrophages. Flourescene intensity of each cytokine in the culture medium was measured by a Multiplex bead-based cytokine assay after 24 h incubation. Normal group (Nor) was treated with media only. Control group (Con) was treated with PIC (the synthetic analog of dsRNA, 50 µg/mL of polyinosinic-polycytidylic acid) alone. IN denotes indomethacin (0.5 µM). Values are the mean ± SD of three independent experiments. ** *P* < 0.01 *vs.* Con; *** *P* < 0.001.

**Figure 4:**
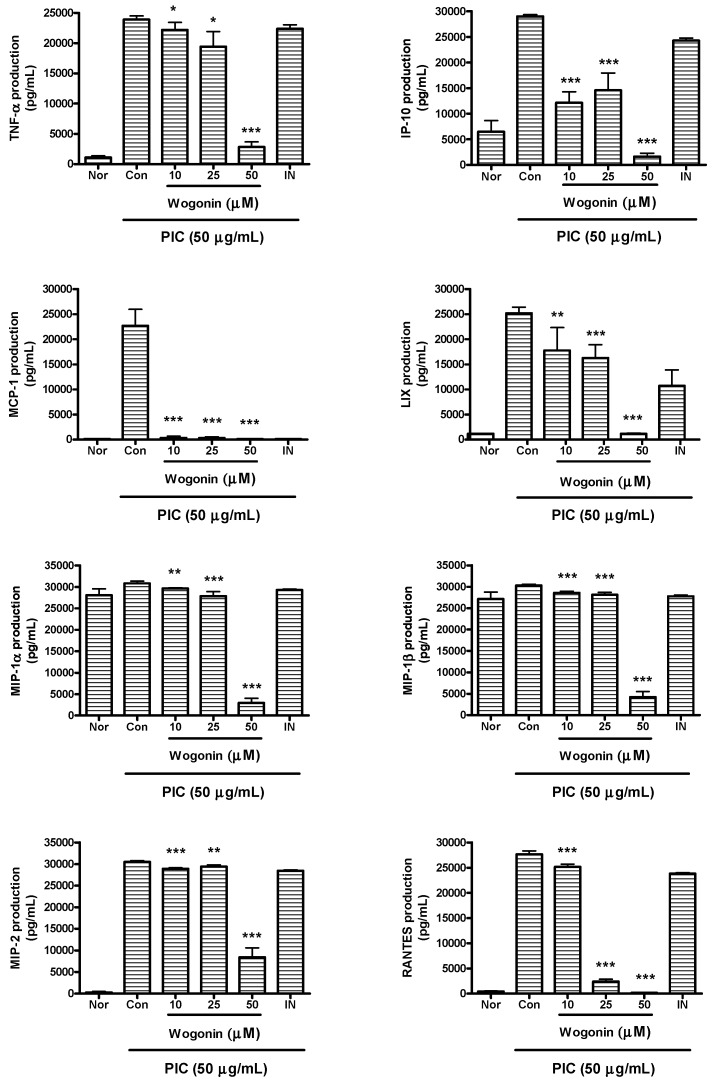
Effects of wogonin on production of cytokines such as TNF-α, IP-10, MCP-1, LIX, MIP-1α, MIP-1β, MIP-2, and RANTES/CCL5 in dsRNA-induced RAW 264.7 mouse macrophages. Flourescene intensity of each cytokine in the culture medium was measured by a Multiplex bead-based cytokine assay after 24 h incubation. Normal group (Nor) was treated with media only. Control group (Con) was treated with PIC (the synthetic analog of dsRNA, 50 µg/mL of polyinosinic-polycytidylic acid) alone. IN denotes indomethacin (0.5 µM). Values are the mean ± SD of three independent experiments. * *P* < 0.05 *vs.* Con; ** *P* < 0.01; *** *P* < 0.001.

Data represented that wogonin significantly reduce excessive productions of IL-1α, IL-1β, IL-6, IL-10, IP-10, G-CSF, GM-CSF, LIF (IL-6 class cytokine), LIX/CXCL5, M-CSF, MCP-1, MIP-1α, MIP-1β, MIP-2, RANTES, TNF-α, and VEGF in PIC-induced RAW 264.7 mouse macrophages (*P* < 0.05) (Figure 3 and Figure 4).

The innate immune response that is normally concerned with host defense against infection can, under some circumstances, cause cell and tissue damage and hence multiple organ failure, the clinical hallmark of sepsis [19]. Weber *et al.* have reported that sepsis could be defined as infection with evidence of systemic inflammation [20]. Sepsis is commonly initiated by infection with various pathogens including viruses, bacteria, fungus, and parasites, but its pathogenesis is characterized by an overwhelming systemic inflammatory response and subsequent immune dysfunction, which can lead to lethal multiple organ failure [12]. Despite the recent advances in intensive care treatment and the discovery of antibiotics, sepsis remains a clinical challenge, with high mortality rates and increasing prevalence [21]. Given the uncontrolled inflammatory pathogenesis of sepsis, anti-inflammatory therapies are used for sepsis management in humans, but have ultimately failed due primarily to sustained immune suppression [22]. Martin *et al.* have reported that sepsis is the second leading cause of death among patients in noncoronary intensive care units of the United States [23]. In the addition, Ulloa and Tracey have reported that severe sepsis remains a major cause of death despite the use of antibiotics [24].

In detail, both viral and bacterial infections contribute to the pathogenesis of severe sepsis, which is characterized by an overwhelming production of proinflammatory cytokines, such as IL-1 and IL-6 [24]. Although IL-1 and IL-6 bring about a valuable inflammatory reaction, the excessive production of IL-1 and IL-6 can be even more dangerous than the original stimulus, causing capillary leakage, tissue injury, and lethal organ failure [25]. IL-10, although traditionally considered as an anti-inflammatory cytokine that modulates the function of adaptive immune-related cells, was also reported to promote abnormal angiogenesis in the eye and in the pathobiology of autoimmune diseases such as lupus and encephalomyelitis [26].

During lung inflammation, various chemokines such as MCP-1, M-CSF, G-CSF, and GM-CSF are increased in bronchoalveolar fluid [27]. MCP-1 and IP-10 are up-regulated within the lung tissue of pneumococcal pneumonia [28].

On the other hand, superfluous or misguided human immune responses may lead to harmful outcomes, as seen in autoimmune disease, chronic inflammation, and allergy [29]. For example, increased IL-6 levels often correlate with several inflammatory autoimmune diseases including RA, systemic-onset juvenile chronic arthritis, osteoporosis, psoriasis, polyclonal plasmacytosis, malignant plasmacytoma, Crohn’s disease, and experimental autoimmune encephalomyelitis [7]. Recently, IL-6 and TNF-α were reported to induce the articular and systemic symptoms of RA [30,31]. The proangiogenic cytokine VEGF in the persistence of inflammatory arthritis supports the hypothesis that expansion of the synovial vasculature is concerned with joint destruction in RA [32]. It was reported that VEGF may be associated with neovascular age-related macular degeneration [33]. Recently, Maskrey *et al.* have reported that chronic inflammation is a typical aspect in vascular diseases, including atherosclerosis [34]. It was also reported that the expression level of MIP-1α, MIP-1β, MIP-2, and RANTES is increased in lung tissue of influenza infection [35]. Recently, it was demonstrated that LIX/CXCL5 expression in bone cells has implications for inflammatory bone diseases such as arthritis and periodontal disease [36]. In psoriasis, a chronic immune-mediated skin disease, the central role of cytokines and their functional interaction with adhesion molecules in the recruitment of tissue-specific lymphocytes has been clearly shown, mainly involving IL-10 and LIF [37]. Concerned with asthma, a chronic inflammatory disease of the airway, it was already reported that bronchial epithelial cell changes in asthma are induced by LIF which promotes the expression of neurokinin-1 receptor, and JAK/STAT pathway and MAPK/ERK pathway may participate in the process [38].

### 2.4. Effects of Wogonin on Intracellular Calcium Release

**Figure 5:**
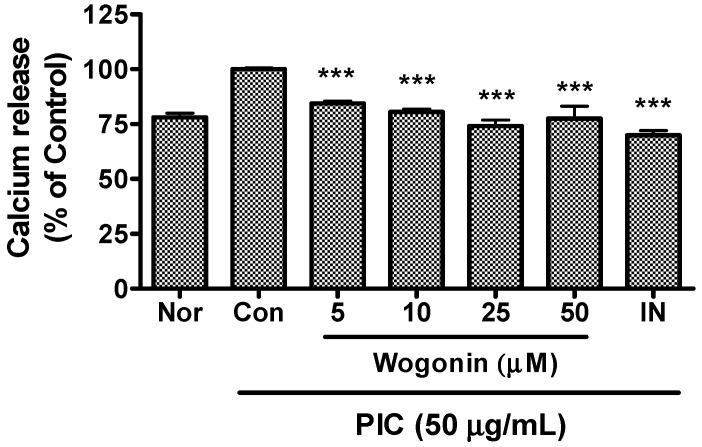
Effects of wogonin on calcium release in dsRNA-induced RAW 264.7 mouse macrophages. After 24 h treatment, calcium release was measured with Fluo-4 calcium assay. Normal group (Nor) was treated with media only. Control group (Con) was treated with the synthetic analog of dsRNA (PIC, 50 µg/mL of polyinosinic-polycytidylic acid alone. IN denotes indomethacin (0.5 µM). Values are the mean ± SD of three independent experiments. *** *P* < 0.001 *vs.* Con.

In the present study, wogonin inhibited the calcium release in PIC-induced RAW 264.7 mouse macrophages (Figure 5). Thus, it can be suggested that wogonin down-regulates excessive production of inflammatory mediators in pathogenic toxicants-induced macrophages through the calcium pathway.

Pathogenic oxidative stress with infection results in macrophage reprogramming with a transient increase of intracellular calcium via the lipid membrane dissociation of the calcium-bound protein annexin VI. This increased cytosolic calcium, in turn, results in the activation of calcium-dependent kinases, leading to enhanced proinflammatory activation [39]. Meanwhile, in oxidative stress, the endoplasmic reticulum (ER) calcium stores are reduced and intracellular calcium concentration is increased, resulting in ER stress-mediated STAT1 activation [40]. 

### 2.5. Effect of Wogonin on mRNA Expression of STAT1 and STAT3

Recently, Filgueiras *et al.* have reported that STAT1 mRNA expression is higher in residential macrophages from autoimmune nonobese diabetic mice compared to nondiabetic mice [41].

**Figure 6:**
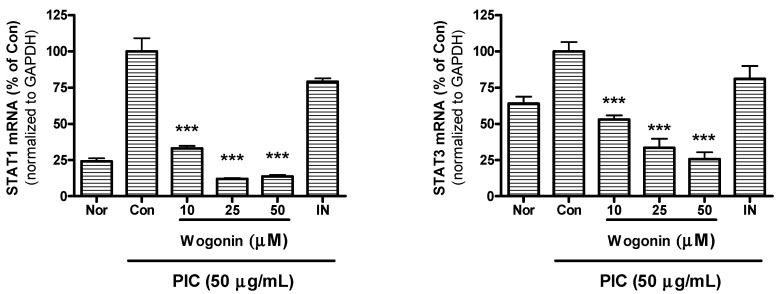
Effect of wogonin on mRNA expression of STAT1 and STAT3 in dsRNA-induced RAW 264.7 mouse macrophages. After 24 h treatment, the mRNA expression of STAT1 and STAT3 was measured by real time RT-PCR assay. STAT1 and STAT3 mRNA were normalized to the housekeeping gene GAPDH. Normal group (Nor) was treated with media only. Control group (Con) was treated with the synthetic analog of dsRNA (PIC, 50 µg/mL of polyinosinic-polycytidylic acid alone. IN denotes indomethacin (0.5 µM). Values are the mean ± SD of three independent experiments. *** *P* < 0.001 *vs.* Con.

In the present study, wogonin significantly inhibited mRNA expression of STAT1 and STAT3 in PIC-induced RAW 264.7 mouse macrophages (Figure 6). The present data suggests that wogonin inhibits calcium release of ER stores and inflammatory reaction in PIC-induced macrophages via calcium-STAT pathway.

## 3. Experimental Section

### 3.1. Materials

DMEM, FBS, penicillin, streptomycin, PBS, and other tissue culture reagents were purchased from Gibco BRL (Grand Island, NY, USA). Wogonin, indomethacin, Griess reagent, and all other chemicals were purchased from Sigma-Aldrich (St. Louis, MO, USA).

### 3.2. Methods

#### 3.2.1. Cell Viability Assay

RAW 264.7 mouse macrophages were obtained from the Korea Cell Line Bank (Seoul, Korea). RAW 264.7 were cultured in DMEM supplemented with 10% FBS containing 100 U/mL of penicillin and 100 µg/mL of streptomycin at 37 °C in a 5% CO_2_ humidified incubator. Cell viability was evaluated with the modified MTT assay.

#### 3.2.2. Quantification of NO Production

NO concentration in culture medium was determined by the Griess reaction. Specifically, after incubation of cells with materials for 24 h, 100 µL of supernatant from each well was mixed with 100 µL of Griess reagent in wells of a 96-well plate. After an incubation of 15 min at room temperature, OD was determined at 540 nm with a microplate reader (Bio-Rad, Hercules, CA, USA).

#### 3.2.3. Multiplex Bead-Based Cytokine Assay

After 24 h treatment with materials, cytokines released from treated cells were measured in cell culture supernatants using a Luminex assay based on xMAP technology. This assay was performed with Milliplex kits (Millipore, Billerica, MA, USA) and Bio-Plex 200 suspension array system (Bio-Rad) as described previously [13,14]. Standard curves for each cytokine were generated using the kit-supplied reference cytokine samples.

#### 3.2.4. Intracellular Calcium Assay

After RAW 264.7 cells were seeded in wells of 96-well plates, materials were added to the culture medium and incubation was carried out for 24 h at 37 °C. Thereafter, the medium was removed and cells were incubated with 100 µL of the Fluo-4 dye loading solution (Molecular Probes, Eugene, OR, USA) for 30 min at 37 °C. After incubation, the fluorescence intensity of each well was determined spectrofluorometrically (Dynex, West Sussex, UK) with excitation and emission filters of 485 nm and 535 nm, respectively.

#### 3.2.5. RNA Isolation and Real Time RT-PCR Analysis

At the end of 24 h incubation with materials, RAW 264.7 mouse macrophages were lysed and Total RNA was isolated using the NucleoSpin RNA kit (Macherey-Nagel, Duren, Germany) according to the manufacturer’s instructions. RNA quantity and quality were confirmed using Experion RNA StdSens Analysis kit (Bio-Rad) and Experion Automatic Electrophoresis System (Bio-Rad). cDNA was synthesized from 1 µg total RNA using iScript cDNA Synthesis kit (Bio-Rad). Real time RT-PCR was performed using the iQ SYBR Green Supermix (Bio-Rad). The GAPDH gene was used for RNA normalization. All samples were run in triplicate. Analysis was performed on a Bio-Rad CFX 96 Real-Time PCR Detection System (Bio-Rad). Relative changes in the gene expression were calculated using the comparative threshold cycle (Ct) method (Bio-Rad).

The following primers were used: STAT1 (GenBank: NM_009283) forward, 5'-TGAGATGTCCCGGATAGTGG-3', reverse, 5'-CGCCAGAGAGAAATTCGTGT-3', STAT3 (NM_213659) forward, 5'-GTCTGCAGAGTTCAAGCACCT-3', reverse, 5'-TCCTCAGTCACGAT CAAGGAG-3', GAPDH (NM_001001303) forward, 5'-AACCTGCCAAGTATGATGAC-3', reverse, 5'-GGGAGTTGCTGTTGAAGT-3'.

#### 3.2.6. Statistical Analysis

The results shown are summarized from three independent experiments and represent the mean ± SD. Significant differences were examined using a Student’s *t*-test with SPSS 11.0 software (SPSS, Chicago, IL, USA). In all cases, a *P* value < 0.05 was considered significant. 

## 4. Conclusions

Although the precise mechanisms regulating the anti-inflammatory activity of wogonin are not yet known, the current study demonstrates that wogonin has anti-inflammatory property related with its inhibition of NO, IL-1α, IL-1β, IL-6, IL-10, IP-10, G-CSF, GM-CSF, LIF (IL-6 class cytokine), LIX/CXCL5, M-CSF, MCP-1, MIP-1α, MIP-1β, MIP-2, RANTES/CCL5, TNF-α, and VEGF in PIC-induced macrophages via calcium-STAT pathway. Actual effect of wogonin on acute and chronic inflammatory diseases deserves to be further studied. 
